# Functional defects in *FOXG1* variants predict the severity of brain anomalies in FOXG1 syndrome

**DOI:** 10.1038/s41380-025-03077-y

**Published:** 2025-06-16

**Authors:** Tsai-Yu Lin, Lee-Chin Wong, Pei-Shan Hou, Chia-Kai Wu, Haw-Yuan Cheng, Hong-Jun Zhao, Chien-Yi Tung, Mei-Hsuan Lee, Wang-Tso Lee, Jin-Wu Tsai

**Affiliations:** 1 Institute of Brain Science, College of Medicine, National Yang Ming Chiao Tung University, Taipei, Taiwan; 2 Laboratory of Molecular Regulation of Neurogenesis, GIGA-Stem Cells and GIGA-Neurosciences, Interdisciplinary Cluster for Applied Genoproteomics (GIGA-R), University of Liège, Liège, Belgium; 3 Department of Pediatrics, National Taiwan University Hospital, Taipei, Taiwan; 4 Department of Pediatrics, National Taiwan University College of Medicine, Taipei, Taiwan; 5 Institute of Anatomy and Cell Biology, College of Medicine, National Yang Ming Chiao Tung University, Taipei, 112 Taiwan; 6 Brain Research Center, National Yang Ming Chiao Tung University, Taipei, 112 Taiwan; 7 Faculty of Medicine, College of Medicine, National Yang Ming Chiao Tung University, Taipei, 112 Taiwan; 8 National Genomics Center for Clinical and Biotechnological Applications of the Cancer and Immunology Research Center, National Yang Ming Chiao Tung University, Taipei, Taiwan; 9 Institute of Clinical Medicine, College of Medicine, National Yang Ming Chiao Tung University, Taipei, 112 Taiwan; 10 Advanced Therapeutics Research Center, National Yang Ming Chiao Tung University, Taipei, Taiwan; 11 Graduate Institute of Brain and Mind Sciences and Department of Pediatrics, National Taiwan University College of Medicine, Taipei, 100 Taiwan; 12 Department of Biological Science and Technology, College of Engineering Bioscience, National Yang Ming Chiao Tung University, Hsinchu, 300 Taiwan

**Keywords:** Neuroscience, Diseases, Genetics

## Abstract

FOXG1 (Forkhead Box G1) is a critical transcription factor for brain development, regulating progenitor cell proliferation, neuronal migration, and cortical circuit assembly. Pathogenic *FOXG1* variants lead to FOXG1 syndrome, a neurodevelopmental disorder characterized by severe brain anomalies and cognitive impairments. Despite efforts to correlate genetic variants with clinical outcomes, the precise relationship remains elusive. Here, we analyzed clinical severity and brain anomalies in 14 individuals with *FOXG1* variants, investigating how these variants impact FOXG1’s properties and functions. We uncovered a strong correlation between the severity of brain anomalies in affected individuals and functional alterations of these variants. Variants with very low protein expression were associated with moderate-to-severe brain anomalies. A luciferase reporter assay was used to assess the ability of FOXG1 variants to repress *COUP-TFI* (*NR2F1*) expression-a function of FOXG1 validated through single-cell RNA-sequencing (scRNA-seq). Variants losing *COUP-TFI* repression ability by binding to *COUP-TFI*’s enhancer region consistently caused moderate-to-severe brain anomalies. Furthermore, *in utero* electroporation (IUE) in embryonic mouse brains was employed to study their impact on neuronal migration and differentiation. Electroporation of wild-type *Foxg1* delayed neuronal migration and altered their cell fate. Remarkably, variants associated with moderate-to-severe brain anomalies impaired these functions, while those with mild brain anomalies caused partial impairment. Thus, by combining protein expression, *COUP-TFI* repression, and neuronal migration assays, we developed a patient stratification paradigm for predicting the severity of FOXG1 syndrome. This workflow successfully differentiated 92.3% of cases, facilitating early diagnosis and guiding future therapeutic interventions.

## Introduction

FOXG1, a transcription factor in the Forkhead (FOX) family, plays a crucial role in brain development [1, 2]. FOXG1 is predominantly expressed in the cerebral cortex, governing multiple developmental processes, such as neural progenitor cell proliferation, neuronal migration, and cell fate determination [3–8]. In early cortical progenitors, FOXG1 induces cell cycle re-entry, upregulates progenitor genes such as *Pax6*, and suppresses Cajal-Retzius cell production [1, 4, 9]. FOXG1’s specific spatial expression pattern and interaction with FGF8 are crucial for cerebral patterning [10, 11]. Additionally, FOXG1 regulates the cell fate of projection neurons by inhibiting the expression of layer VI gene *Tbr1*, leading to de-repression of the expression of layer V gene *Ctip2*, thus facilitating the transition from producing layer VI neurons to layer V neurons [12–14]. Later, FOXG1 downregulation in young neurons causes the de-repression of *COUP-TFI* (also known as *NR2F1*), leading to the production of layer IV sensory recipient neurons from SATB2-expressing upper-layer projection neurons [7]. These findings underscore FOXG1’s diverse roles in cerebral cortex development and the potential impacts of FOXG1 mutations.

Heterozygous variants of *FOXG1* can lead to FOXG1 syndrome, a rare neurodevelopmental disorder previously described as a congenital form of Rett syndrome [15], with an estimated incidence of 1 in 30,000 [16]. Individuals with FOXG1 syndrome may exhibit microcephaly, simplified gyral patterns, underdeveloped frontal cortex, and hypogenesis or agenesis of the corpus callosum [17, 18]. They also suffer from severe intellectual disability, epilepsy, hyperkinetic movement disorders, sleep disorders, and deficits in language and motor skills [19, 20]. Genetically, FOXG1 syndrome involves de novo mutations, primarily loss-of-function (LOF) variants resulting in FOXG1 haploinsufficiency [21], while a few cases with duplication of *FOXG1* may lead to elevated FOXG1 levels [22]. Pathogenic variants impact various FOXG1 protein domains, leading to diverse clinical manifestations [17–20, 23]. Specific genotypes, such as truncating variants in the N-terminus, tend to be associated with more severe clinical features and brain anomalies, while milder phenotypes are associated with missense variants in the Forkhead DNA binding domain (FBD) [18–20]. However, this relationship is not absolute, as certain genotypes may exhibit variable clinical and imaging characteristics [17, 19]. The genetic variability among patients poses a challenge in dissecting genotype-phenotype associations [24].

Despite advances in next-generation sequencing (NGS), determining the impact of identified variants remains challenging [25], as a healthy individual’s exome contains numerous variations compared to the standard genome. Various databases and tools, including gnomAD [26] (https://gnomad.broadinstitute.org/), SIFT [27] (https://sift.bii.a-star.edu.sg/), PolyPhen [28] (http://genetics.bwh.harvard.edu/pph2/), and VEP [29] (https://www.ensembl.org/vep), assist in identifying potential disease-causing variations, though the methodology is subject to ongoing refinement. Importantly, functional assays capable of distinguishing between benign and pathogenic variants in many genetic diseases, including FOXG1 syndrome, are still lacking.

This study investigates how pathogenic *FOXG1* variants with different severities impact the properties and functions of the protein, particularly in cortical development. We uncovered a strong correlation between the severity of brain anomalies and functional alterations. Consequently, we have established a workflow of function assays for predicting brain anomalies and clinical severity resulting from *FOXG1* variants.

## Materials and methods

### Recruitment of FOXG1 syndrome cases

We enrolled individuals with genetically confirmed FOXG1 syndrome in our hospital and integrated data from previously reported cases from the literature to investigate the impact of FOXG1 variants in different domains. Inclusion criteria were: (1) a pathogenic or likely pathogenic intragenic *FOXG1* variant defined by American College of Medical Genetics and Genomics (ACMG) guidelines, and (2) comprehensive clinical descriptions with cranial MRI scans (Supplementary Fig. 1).

### Clinical characterization

We used the FOXG1 syndrome clinical severity scoring system [19] to assess the impact on overall functioning and daily life systematically. The scoring system included 17 phenotypic items across four categories: somatic growth (4 items), motor and speech development (4 items, if applicable), behavior (3 items), and neurological features (6 items). Each item was rated on a 0 to 2-point scale, and the clinical severity score (CSS) was the average of these items, ranging from 0–2 [19]. Severity was classified as mild (0–0.67), moderate (0.68–1.33), and severe (1.34–2).

### Neuroimaging characterization

Brain MRI images were analyzed by two independent pediatric neurologists, W.-T. Lee and L.-C. Wong, and scored using the FOXG1 brain MRI severity score (MRI SS), adapted from M. Pringsheim et al. [18] (Supplementary Table 1). This scoring system assessed critical factors like simplified gyral patterns, basal ganglia hypoplasia, enlargement of inner CSF spaces, anomalies in the corpus callosum, and frontal lobe hypoplasia. Each of these items was assigned a rating scale, with scores ranging from 0–1 or 0–3, depending on the specific item under consideration. Total scores ranged from 0–6, with severity levels categorized as normal (0), mild (1–2), moderate (3–4), and severe (5–6).

### Constructs

The human *FOXG1* (NM_005249.5) and mouse *Foxg1* (NM_001160112.1) are highly conserved, sharing 93.74% similarity. To ensure compatibility with mouse experiments, we constructed a plasmid containing mouse *Foxg1* cDNA tagged with Myc-tag at its N-terminus, based on pCMV and pCAG vectors. To create the FOXG1 syndrome-associated variants, mutagenesis was performed following the manufacturer’s instructions using QuikChange II Site-Directed Mutagenesis Kit. Sequence alignment was performed using the protein BLAST algorithm (National Institutes of Health, USA).

### Western blotting

Cells were lysed using RIPA buffer containing 10% protease and sonicated. Following sonication, the lysate rotated at 4 °C for 1 h on a rotor. The supernatant was collected after centrifugation at 15,000 g for 15 min at 4 °C. Protein concentrations were determined using the BCA assay (Thermo Fisher Scientific, USA). Proteins were separated by electrophoresis on a 0.1% SDS and 10% acrylamide gel, transferred to a PVDF membrane, and probed with primary antibodies: anti-BF-1 (Takara; diluted 1:5000), anti-Myc (Thermo Fisher Scientific; diluted 1:5000), and mouse anti-GAPDH (GeneTex; diluted 1:5000). The primary antibodies were detected with horseradish peroxidase (HRP)-conjugated secondary antibodies: anti-mouse (GeneTex; diluted 1:10000) and anti-rabbit (Sigma Aldrich; diluted 1:10000). Signals were developed with ECL reagent (Millipore) and detected by Luminescence/Fluorescence Imaging system Amersham Imager 680 (GE). Protein band intensities were analyzed using ImageJ software (NIH, USA). Three independent experiments from three batches of transfected cells were performed.

### Immunofluorescence staining of cultured cells

HEK293T cells (60,019), purchased from BCRC, were fixed in 4% paraformaldehyde, washed with phosphate-buffered saline (PBS), and permeabilized in 0.1% in PBST at 4 °C for 15 min, followed by blocking at room temperature for 20 min in PBS with 2% bovine serum albumin and 4% normal goat serum. Subsequently, cells were incubated for up to 2 h or overnight with primary antibodies: anti-BF-1 (Takara; diluted 1:500) for detecting FOXG1 and anti-Myc (Thermo Fisher Scientific; diluted 1:500) for detecting Myc-tag. After PBS washes twice, cells were incubated in secondary antibodies: anti-mouse (GeneTex; diluted 1:10000) and anti-rabbit (Sigma Aldrich; diluted 1:10000) for 1 h, followed by a 15-min DAPI staining. Images were acquired using a confocal microscope (Zeiss LSM700, Germany).

### scRNA-seq analysis

For mouse gene profiling, we utilized our previously published scRNA-seq dataset from C57BL/6 embryonic mouse brains at embryonic day (E) 13.5 and E15.5 of mixed sex [30]. Additionally, we analyzed scRNA-seq data from CD-1 mouse embryos, also of mixed sex, spanning developmental stages E10, E12, E14, E15, E16, and E18 [31]. For human gene profiling, we employed scRNA-seq data from the future somatosensory area of the human embryonic forebrain at gestational week (GW) 18 [32], including samples of mixed sex. Analysis of single cell transcriptomics was performed using Seurat, an R-based toolkit [33]. Cells from the dorsal pallium were selected based on the expression of neocortical markers, such as *EMX1* and *EMX2*. Cell identity, including progenitor, precursor, and neuron populations, was determined through cell cycle scoring using Seurat’s built-in functions, alongside the expression of key markers, including progenitor genes such as *Pax6*/*PAX6*, precursor genes such as *Tbr2*/*TBR2*, and neuronal genes such as *Nex*/*NEX*.

### Luciferase reporter assay

The luciferase reporter assay followed the methods in previous reports [7, 34]. Briefly, the *COUP-TFI* gene was cloned into pGL4.1 with or without FOXG1 binding site PBS1. pRL-TK, containing the Renilla luciferase, served as an internal standard for transfection efficiency. U87-MG cells (Food Industry Research and Development Institute, Taiwan) were co-transfected with reporter plasmid (0.5 µg) and pRL-TK (0.05 µg) using Lipofetamine 3000 (Invitrogen). After 48 h, cells were harvested for luciferase activity measurement by the Dual-Luciferase Assay System (Promega). Signals were measured using TECAN 200/200Pro. Relative luciferase units (RLUs) were calculated by normalizing luciferase activity to Renilla activity, which was further normalized to cells with the reporter plasmid containing *COUP-TFI* promoter. All values of luciferase assay were normalized by pGL4.1, and *FOXG1* variants were further normalized by WT *FOXG1*. Three independent experiments from three batches of transfected cells were performed.

### *In utero* electroporation (IUE)

IUE was performed according to previous studies [34–38]. Briefly, *Foxg1* (1 μg/μl) and GFP constructs (0.5 μg/μl) were co-injected into the lateral ventricle of embryonic brains of ICR mice (BioLASCO) at E13.75 and electroporated with a diameter 5 mm forceps electrode, which transmitted five electric pulses at 40 V for 50 ms at 1-s interval through the uterine wall by an electroporation generator (Harvard Apparatus). Brains of the electroporated embryos of mixed sex were harvested after the electroporation at E16.75 or P7 and subsequently perfused with PBS and 4% paraformaldehyde (PFA) for tissue fixation. Three independent experiments from three animals for each group were performed. The order of injection was randomized and blinded in each IUE experiment.

### Immunofluorescence staining of brain slices

Brain slices were permeabilized in PBST (PBS with 0.2% Triton X-100) for 30 min. After blocking with 10% normal goat serum/5% BSA/0.2% PBST solution, slices were incubated with the primary antibody at 4 °C for 36 h. Slices were then incubated with the secondary antibody at room temperature for 2 h, and stained with DAPI for 1 h. Stained brain slices were mounted in Prolonged Gold Antifade reagent (Life Technologies, USA) and stored at 4 °C until imaging, all handled in darkness. Images were acquired using a confocal microscope (Zeiss LSM700, Germany) with 5X and 20X objectives and analyzed with ImageJ FIJI software (NIH, USA). Primary antibodies used include rabbit anti-TBR1 (ab31940, Abcam), rabbit anti-BF1 (M227, Takara), mouse anti-RORβ (N7927, R&D), and mouse anti-BRN2 (sc-393324, Santa Cruz Biotechnology).

### Statistical analysis

For statistical analysis, Prism (GraphPad) was used to conduct One- and Two-way ANOVA. Dunnett’s multiple comparisons test and uncorrected Fisher’s LSD are used as post-hoc tests. Data were shown as the mean ± S.E.M. All data were obtained from 3 independent experiments. A significant difference was defined as *p* < 0.05.

## Results

### Clinical, genetic, and image characteristics in individuals with FOXG1 syndrome

**Fig. 1:**
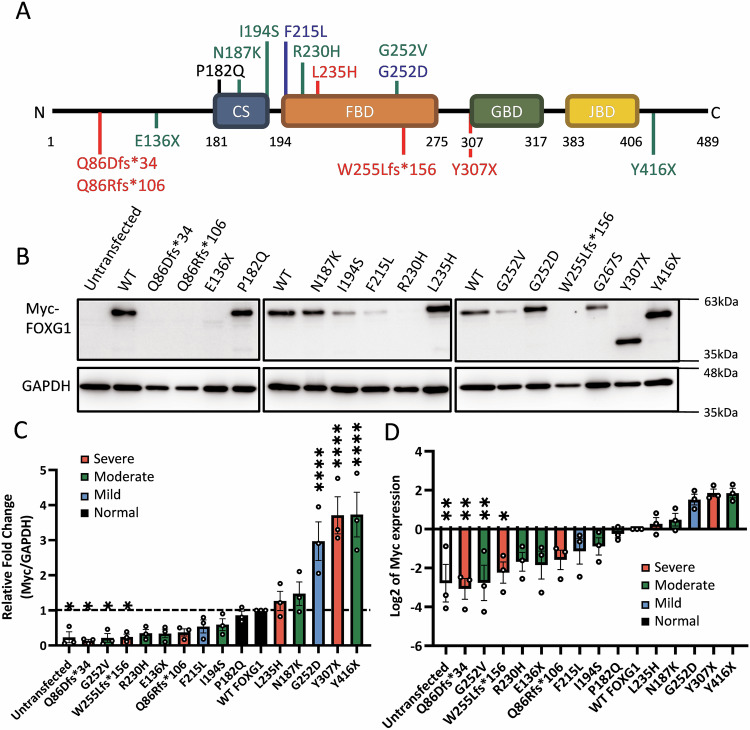
Variant locations in *FOXG1* and their expression levels. **A** Localization of the 14 *FOXG1* variants analyzed in this study. Three variants lead to early termination at the N terminus, and three variants result in truncation at the C terminus, while the remaining 8 variants are missense variants within or in proximity to the CS and FBD domains. Variants causing different severities of brain anomalies are color-coded: Red for severe, green for moderate, blue for mild, and black for normal. **B** Protein expression of FOXG1 and its variants in transfected cells, determined by western blotting with Myc antibody. GAPDH served as the loading control. **C** Bar graph showing the relative expression of FOXG1 variants compared to WT FOXG1 in ascending order of expression (*n* = 3 independent transfections). **D** Bar graph presenting the relative expression of FOXG1 variants on a log_2_ scale (*n* = 3 independent transfections). Error bars: S.E.M. *p < 0.05, **p < 0.01, ****p < 0.0001. One-way ANOVA; post-hoc: Uncorrected Fisher’s LSD.

We collected clinical, brain MRI, and genetic data from 14 FOXG1 syndrome cases, with 5 from our hospital and 9 from the literature [17, 18, 39, 40]. All cases carried intragenic heterozygous *FOXG1* variants, including p.Gln86AspfsTer34 (Q86Dfs*34), p.Gln86ArgfsTer106 (Q86Rfs*106), p.Glu136Ter (E136X), p.Pro182Gln (P182Q), p.Asn187Lys (N187K), p.Ile194Ser (I194S), p.Phe215Leu (F215L), p.Arg230His (R230H), p.Leu235His (L235H), p.Gly252Val (G252V), p.Gly252Asp (G252D), p.Trp255LeufsTer156 (W255Lfs*156), p.Tyr307Ter (Y307X), and p.Tyr416Ter (Y416X) (Table 1). These variants covered different FOXG1 domains (Fig. 1A), including 3 N-terminal early terminations (2 frameshift and 1 nonsense), 9 variants within or near the FBD and CS domains (8 missense and 1 frameshift), and 2 C-terminal nonsense variants (Table 1, Fig. 1A).

**Table 1:** Genetic and phenotypic features of individuals with FOXG1 syndrome.

No.	Genotype (ref.)	Amino acid change	Coding Effect	FOXG1 Domain	Sex	Age (month)	Microcephaly	Clinical Severity	CSS	Age at MRI (month)	Brain MRI severity	MRI SS
1	c.256dup C^A^	Q86Dfs*34	Frameshift	N-terminal	M	54	Yes	Moderate	1.06	11	Severe	5
2	c.256del C^A^	Q86Rfs*106	Frameshift	N-terminal	M	263	Yes	Severe	1.56	84	Severe	6
3	c.406 G > T [18]	E136X	Nonsense	N-terminal	M	33	Yes	Severe	1.44	7	Moderate	4
4	c.545 C > A [18]	P182Q	Missense	FBD, CS	F	85	Yes	Moderate	0.71	24	Normal	0
5	c.561 C > A [18]	N187K	Missense	FBD, CS	F	62	None	Severe	1.60	12	Moderate	3
6	c.581 T > G [18]	I194S	Missense	FBD, CS	F	72	Yes	Moderate	1.25	10	Moderate	4
7	c.645 C > A^A^	F215L	Missense	FBD	F	84	Yes	Mild	0.56	42	Mild	1
8	c.689 G > A [40]	R230H	Missense	FBD	F	96	Yes	Moderate	1.00	96	Moderate	4
9	c.704 T > A^A^	L235H	Missense	FBD	M	16	Yes	Moderate	0.79	9	Severe	6
10	c.755 G > T [17]	G252V	Missense	FBD	F	108	Yes	Moderate	0.75	19	Moderate	4
11	c.755 G > A [18]	G252D	Missense	FBD	F	192	Yes	Severe	1.47	192	Mild	1
12	c.763_893del^A^	W255Lfs*156	Frameshift	FBD	M	49	Yes	Moderate	1.31	22	Severe	6
13	c.921 C > G [18]	Y307X	Nonsense	C-terminal	F	33	Yes	Moderate	1.29	6	Severe	5
14	c.1248 C > G [39]	Y416X	Nonsense	C-terminal	F	60	Yes	Moderate	1.20	22	Moderate	4

Clinical severity was determined by using the clinical severity score (0–2), where a score of 0–0.67 indicated mild severity, a score of 0.68–1.33 indicated moderate severity, and a score of 1.34–2 indicated severe severity. Regarding brain MRI severity, the MRI SS ranged from 0–6, where a score of 0 indicated completely normal MRI results and a score of 6 indicated the most severe neuroimaging anomaly.

^A^Our cases.

The predominant clinical manifestation was microcephaly, affecting 13 out of 14 cases. A comprehensive analysis of brain MRIs (Table 1, Supplementary Fig. 1) revealed distinct neuroimaging features, with anomalies in the corpus callosum (11/14) and a simplified gyral pattern (11/14) being the most prevalent. This was followed by hypoplasia of the frontal lobes (9/14), enlargement of inner CSF spaces (8/14), and hypoplasia of the basal ganglia relative to the thalami (7/14). Anomalies within the corpus callosum were further categorized as partial agenesis in 7 individuals and thinning in 4 individuals.

Clinical severity ranged from mild to severe, with N-terminal variants presenting moderate to severe clinical severity, while those with pathogenic variants within the FBD domain exhibited heterogeneous severity, ranging from mild to severe. Individuals with variants within the C-terminal region displayed moderate clinical severity. The brain MRI severity score, reflecting the summation of brain anomalies, also ranged from mild to severe (Table 1). Interestingly, N-terminal variants correlated with severe brain MRI anomalies, whereas FBD domain variants exhibited heterogeneous severity. Variants in the C-terminal region were linked to moderate-to-severe brain anomalies. Although there was some correlation between variant location and clinical features, they did not provide a perfect prediction of severity.

### Low-expressing *Foxg1* variants are linked to moderate-to-severe brain anomalies

To investigate the effects of different variants on FOXG1 expression, we cloned and transfected Myc-tagged *Foxg1* (pCMV-Myc-Foxg1) into U87-MG human glioblastoma cells. The FOXG1 protein localized correctly within the nucleus (Supplementary Fig. 2). Subsequently, disease-associated variants were generated and transfected into U87-MG cells, and protein expression was analyzed after two days in culture (DIV) by western blotting (Fig. 1B). Among the nonsense variants, E136X exhibited very low protein expression, whereas Y307X and Y416X showed higher expression compared to wild-type (WT) FOXG1. For missense variants, I194S, F215L, R230H, and G252V displayed lower expression, while others (P182Q, N187K, L235H, and G252D) exhibited levels similar to or higher than WT FOXG1. All frameshift variants (Q86Dfs*34, Q86Rfs*106, W255Lfs*156) consistently displayed very low expression (Fig. 1B–D).

Protein expression levels were categorized as very low (Q86Dfs*34, Q86Rfs*106, E136X, R203H, G252V, W255Lfs*156), low (I194S, F215L), normal (P182Q, N187K, L235H), and high (G252D, Y416X, Y307X). Interestingly, variants with very low expression correlated with moderate-to-severe MRI findings, suggesting a link between reduced expression and severe phenotypes. This assay alone identified 54.5% (6/11) of cases with moderate-to-severe brain anomalies. However, due to its low correlations with the brain MRI and clinical severity scores (R^2^ = 0.0072 and 0.046, respectively; Supplementary Fig. 3A), additional assays are necessary for a more accurate prediction of FOXG1 syndrome severity.

### Failure to repress the *COUP-TFI* promoter is associated with moderate-to-severe brain anomalies

**Fig. 2:**
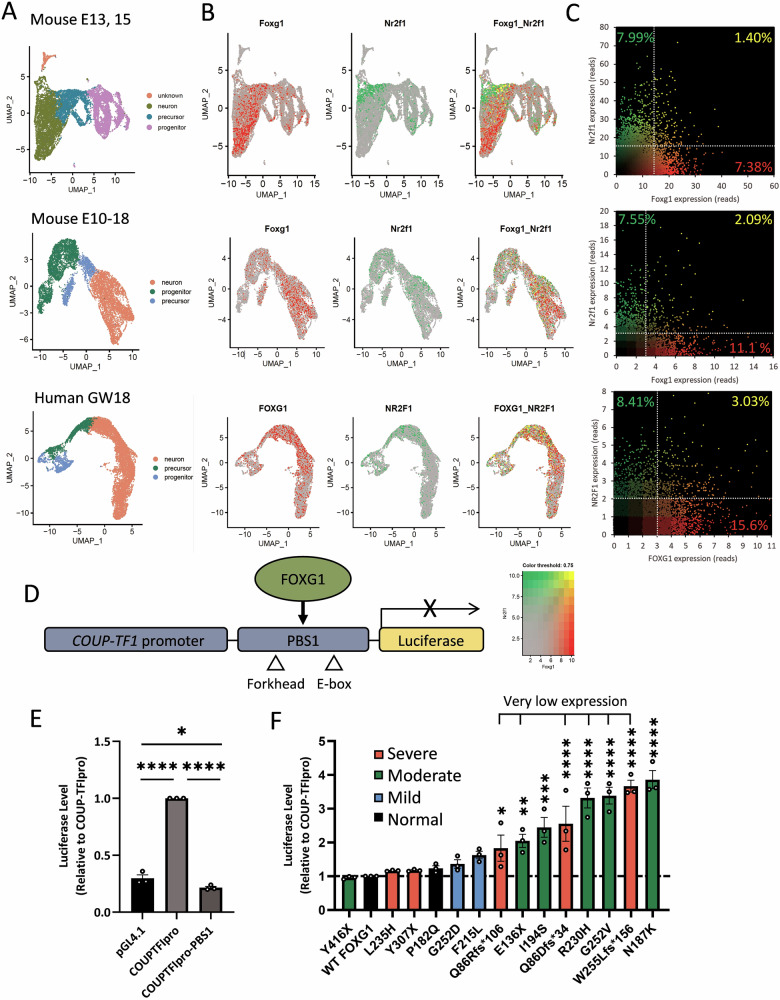
Expression of *Foxg1* and *COUP-TFI* (*Nr2f1*) in the developing cerebral cortex and the *COUP-TFI* repression ability of FOXG1 variants. **A** UMAP plots depicting scRNA-seq results of the cortices from mouse embryos at E13.5 and E15.5 (top); mouse embryos at E10, 12, 14, 15, 16 and 18 (middle); and human fetus at GW18 (bottom). Three developmental states (progenitors, precursors, and neurons) are identified based on their marker genes. **B** Heat maps illustrating the expression of *Foxg1*/*FOXG1* (red) and *COUP-TF1* (*Nr2f1*/*NR2F1*, green), revealing distinct expression patterns. *Foxg1*/*FOXG1* and *Nr2f1*/*NR2F1* expression exhibit mutually exclusive expression patterns. **C** Scatter plots of *Foxg1*/*FOXG1* and *Nr2f1*/*NR2F1* expression in these cells shows relatively few cells co-expressing *Foxg1*/*FOXG1* and *Nr2f1*/*NR2F1*. **D** Diagram outlining the luciferase reporter assay. Luciferase expression is driven by the *COUP-TFI* promoter (*COUPTF1pro*) and its enhancer-like region PBS1. Co-transfection of *Foxg1* and the reporter construct allows FOXG1 to bind to the PBS1 region, repressing the expression of downstream genes. **E** Verification of WT FOXG1 repression of *COUP-TFI*, requiring PBS1. Cells transfected with *Foxg1* along with the Luciferase reporter driven by *COUPTF1pro* without PBS1 exhibited high luciferase activity. In contrast, the inclusion of PBS1 reduced the luciferase activity, confirming a repression function of FOXG1 (*n* = 3 independent transfections). **F** Luciferase activity of cells transfected with *Foxg1* variants along with the luciferase reporter driven by the *COUP-TFI* promoter and PBS1. While the expression of Y416X, L235H, Y307X, P182Q, G252D, and F215L resulted in similarly low luciferase activity as WT FOXG1, the expression of other variants showed significantly higher luciferase activity, indicating impaired COUP-TFI repression function (*n* = 3 independent transfections). Error bars: S.E.M. *p < 0.05, **p < 0.01, ***p < 0.001. ****p < 0.0001. One-way ANOVA; post-hoc: Uncorrected Fisher’s LSD.

Given *FOXG1*’s established role in repressing *COUP-TFI* (*NR2F1*) expression [7], we analyzed *Foxg1* and *Nr2f1* expression using our scRNA-seq data from mouse cortices at E13.5 and E15.5 [30]. Additionally, scRNA-seq data from mouse embryos spanning E10–E18 were examined [31] (Fig. 2, Supplementary Fig. 4). Cells were categorized into three main types: progenitor (i.e., radial glial cells), precursor (i.e., intermediate progenitors), and neuron, based on various cell markers, including *Pax6*, *Emx1*, *Eomes*, *Neurod6*, *Tbr1*, *Bcl11b*, *Satb2*, and *Fabp7* (Fig. 2A) [30]. We found that, at both stages, *Foxg1* was predominantly expressed in neurons, with minor expression in progenitors and precursors, while *Nr2f1* showed neuron-specific expression (Fig. 2B). Interestingly, *Foxg1* and *Nr2f1* expression appeared mutually exclusive (Fig. 2C), consistent with *Foxg1*’s role in repressing *Nr2f1* expression during cortical development [7]. We extended this analysis using scRNA-seq data from the human embryonic forebrain at GW18 [32], where we observed a similar mutual exclusion between *FOXG1* and *NR2F1*, confirming the conserved nature of this regulatory mechanism between mouse and human cortical development.

To assess the impact of FOXG1 variants on the ability to repress *COUP-TFI* expression, we used a luciferase reporter construct with the *COUP-TFI* promoter and *COUP-TFI* enhancer PBS1 (Fig. 2D). Previous studies showed FOXG1 binding to PBS1 represses the promoter activity, downregulating *COUP-TFI* [7]. In this “*COUP-TFI* repression assay,” the reporter construct and *Foxg1* cDNA were co-transfected into U87-MG cells. WT FOXG1 led to low luciferase activity, consistent with its repressor role in *COUP-TFI* expression (Fig. 2E) [7].

Comparing luciferase luminescence among cells expressing FOXG1 variants, 4 variants (P182Q, L235H, Y307X, and Y416X) showed low activities similar to WT FOXG1 (Fig. 2F). In contrast, G252D and F215L, associated with mild MRI severity, exhibited slightly increased activity, suggesting partial LOF. The remaining 8 FOXG1 variants, associated with moderate (E136X, N187K, I194S, R230H, G252V) or severe (Q86Dfs*34, Q86Rfs*106, W255Lfs*156) MRI severity scores, displayed elevated activity, indicating LOF in *COUP-TFI* repression. Notably, all 6 variants with very low protein expression exhibited significantly higher luciferase activity compared to WT FOXG1 (*p* < 0.05, Fig. 2F), consistent with expected LOF effects. Among other variants, only N187K and I194S showed LOF in the repression of luciferase activity, both associated with moderate MRI severity, regardless of their expression levels (N187K: normal; I194S: low). While correlations between *COUP-TFI* repression and the brain MRI (R^2^ = 0.085) and clinical severity (R^2^ = 0.090) scores remained low (Supplementary Fig. 3B), this additional assay increased sensitivity in detecting variants causing moderate-to-severe brain anomaly from 54.5% (6/11) to 72.7% (8/11).

### Impact on neuronal migration correlates with brain MRI severity

**Fig. 3:**
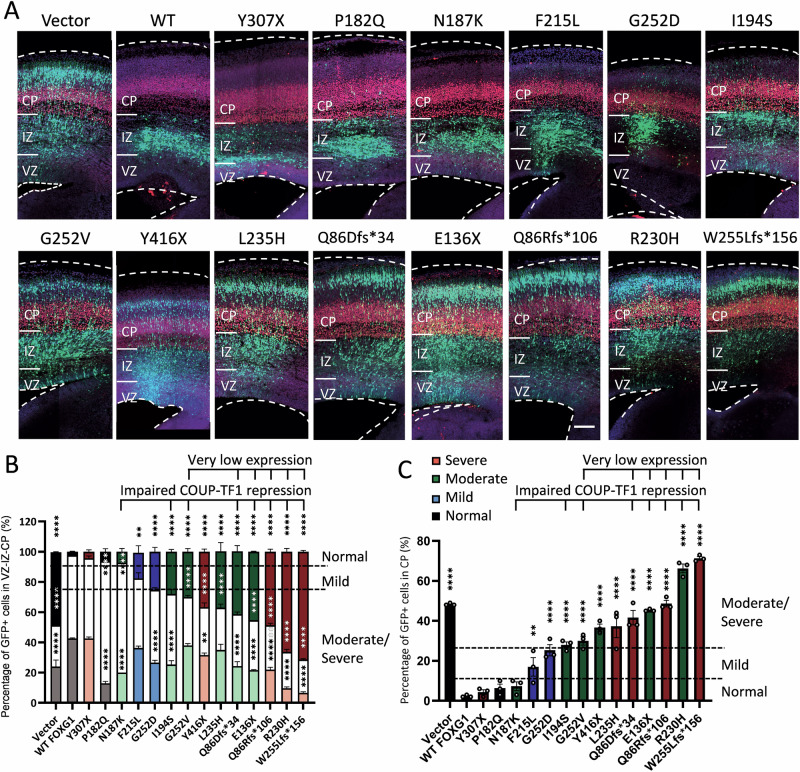
Effects of overexpression of FOXG1 WT and variants on neuronal migration by IUE. **A** Neuronal distribution of brains electroporated with Foxg1 and the pathogenic variants. Mouse brains were electroporated with GFP along with Foxg1 WT or its variants at E13.75. Neuronal cell distribution was examined 3 days after IUE in brain slices stained with the layer marker TBR1 (red) and DAPI (blue). While more than half of the cells electroporated with the empty vector migrated to the CP, FOXG1 overexpression altered neuronal migration, causing most cells to accumulate in the VZ and IZ. Cells electroporated with pathogenic *Foxg1* variants displayed varying degrees of migration alteration. Bar = 100 μm. **B** The bar graph shows cell distributions in the VZ, IZ, and CP after IUE. **C** The bar graph shows cell distributions in the CP after IUE. Dashed horizontal lines indicate 10 and 25% of cells distributed in the CP. Error bars: S.E.M. *p < 0.05, **p < 0.01, ***p < 0.001. ****p < 0.0001. Two-way ANOVA, post-hoc: Dunnett’s multiple comparisons test (*n* = 3 independent IUE experiments).

Previously, FOXG1 overexpression in the developing mouse cortex at E13.75 delayed neuronal migration and attenuated layer IV neuronal fate [7]. To investigate how *FOXG1* variants impact these functions, we used IUE to introduce constructs overexpressing WT FOXG1 or its variants, along with GFP plasmid, into neural progenitors at E13.75 (Fig. 3). Three days post-IUE, brain sections stained with TBR1 (T-Box Brain Transcription Factor 1) as a marker for the deep cortical plate (CP) showed that, in control brains electroporated with the empty vector, about half of the GFP+ cells had migrated from the ventricular zone (VZ) to the CP, with some cells in transit within the intermediate zone (IZ) and VZ. Notably, upon electroporation of WT *Foxg1*, most electroporated GFP+ cells were distributed in the IZ and VZ, with very few cells (<10%) reaching the CP, consistent with previous studies [7].

IUE of disease-associated *FOXG1* variants showed varied effects (Fig. 3). Brains electroporated with 6 variants with very low expression (Q86Dfs*34, Q86Rfs*106, E136X, R203H, G252V, W255Lfs*156) showed many GFP+ cells in the CP (>25%), similar to brains electroporated with the vector only, indicating functional impairment. Among the two other variants showing LOF in *COUP-TFI* repression, I194S overexpression also resulted in > 25% of cells reaching the CP. Curiously, N187K overexpression led to very few cells reaching the CP (<10%), suggesting retained functionality in affecting neuronal migration. Of the remaining 6 variants without LOF in *COUP-TFI* repression, P182Q and Y307X exhibited similar percentages of cells in the CP compared to WT *Foxg1* (<10% in all 3 groups, *p* > 0.05), suggesting relatively normal function. F215L and G252D resulted in 10–25% of cells migrating to the CP, suggesting some LOF. L235H and Y416X led to > 25% of GFP+ cells in the CP, suggesting a greater LOF. Collectively, our findings reveal a good correlation between functional impairment in these variants and the severity of brain anomalies (R^2^ = 0.405, Supplementary Fig. 3C). Combining these three functional assays increased the prediction rate for variants causing moderate-to-severe brain anomaly to 90.9% (10/11), except for the Y307X variant, which, despite minimal LOF in all three assays, was associated with severe brain anomalies.

### Correlation between brain MRI severity and altered cell fate

**Fig. 4:**
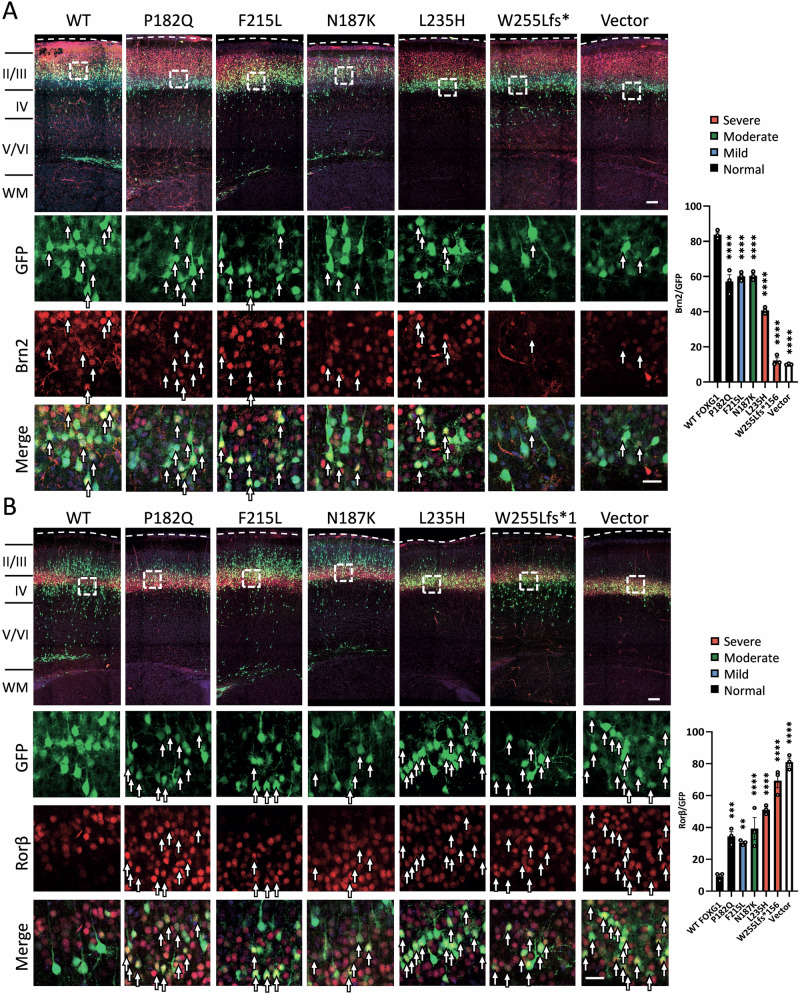
Effects of the expression of FOXG1 and its variants on the expression of the layer II/III and IV neuronal markers in the developing cortex. Mouse brains were electroporated with GFP (green) along with *Foxg1* variants or the empty vector at E13.75. Brain sections at P7 were immunostained with the layer II/III marker BRN2 or layer IV marker RORβ, and counterstained with DAPI (blue). **A** Neurons electroporated with the empty vector were mostly BRN2- and distributed in layer IV, while FOXG1-expressing neurons were redistributed to layer II/III and expressed BRN2 (red). Neurons expressing pathogenic *Foxg1* variants displayed variable effects on this fate change. The lower panels show high-magnification images with separated channels. Arrows indicate BRN2+ neurons. Bars = 100 μm. The bar graph shows the percentage of BRN2+ neurons among GFP+ cells after overexpressing *Foxg1* WT and its variants. The percentage of BRN2+ cells inversely correlates with the severity of brain malformations. **B** Conversely, most neurons electroporated with the empty vector were RORβ+ (red), while neurons expressing FOXG1 were redistributed to layer II/III and became RORβ-. Neurons expressing pathogenic variants displayed varying effects on this fate change. Lower panels show the high-magnification images with separated channels. Arrows indicate RORβ+ neurons. Bars = 100 μm. The bar graph shows the percentage of RORβ+ among GFP+ cells after overexpressing FOXG1 WT and its variants. The percentage of RORβ+ cells correlates well with the severity of brain malformations. Error bars: S.E.M. *p < 0.05, **p < 0.01, ***p < 0.001. ****p < 0.0001. One-way ANOVA, post-hoc: Uncorrected Fisher’s LSD (*n* = 3 independent IUE).

To investigate the impact of FOXG1 variants on neuronal cell fate, brains after IUE at E13.75 were stained with layer-specific markers at P7, including BRN2 (Brain-specific homeobox/POU domain protein 2) for layer II/III and RORβ (Retinoic acid-related orphan receptor beta) for layer IV (Fig. 4). In control brains, most GFP+ cells reached layer IV and expressed RORβ but not BRN2. In contrast, FOXG1 overexpression shifted cells to layers II/III, V, and VI, predominantly RORβ- and BRN2+, consistent with a mutually repressive interaction between RORβ and BRN2 [41].

Brains electroporated with *Foxg1* variants causing normal (P182Q), mild (F215L), and moderate (N187K) brain anomalies showed a slight but significant reduction in BRN2+ cells compared to *Foxg1* WT-electroporated brains (Fig. 4), indicating some LOF in altering neuronal differentiation. Notably, brains electroporated with variants leading to severe brain anomalies (L235H and W255Lfs*156) exhibited even greater reductions in BRN2+ cells, suggesting disrupted FOXG1 function. Conversely, brains electroporated with normal-to-moderate variants (P182Q, F215L, and N187K) showed partial LOF in directing neurons from RORβ+ to RORβ-, whereas severe variants (L235H and W255Lfs*156) lost most of this function (Fig. 4). Although we assessed only a subset of the 14 variants spanning different severity levels due to the complexity of this assay, these results suggest a correlation between the ability to alter cell fate and the severity of brain anomalies in FOXG1 syndrome.

### Clinical severity prediction workflow for FOXG1 syndrome

**Fig. 5:**
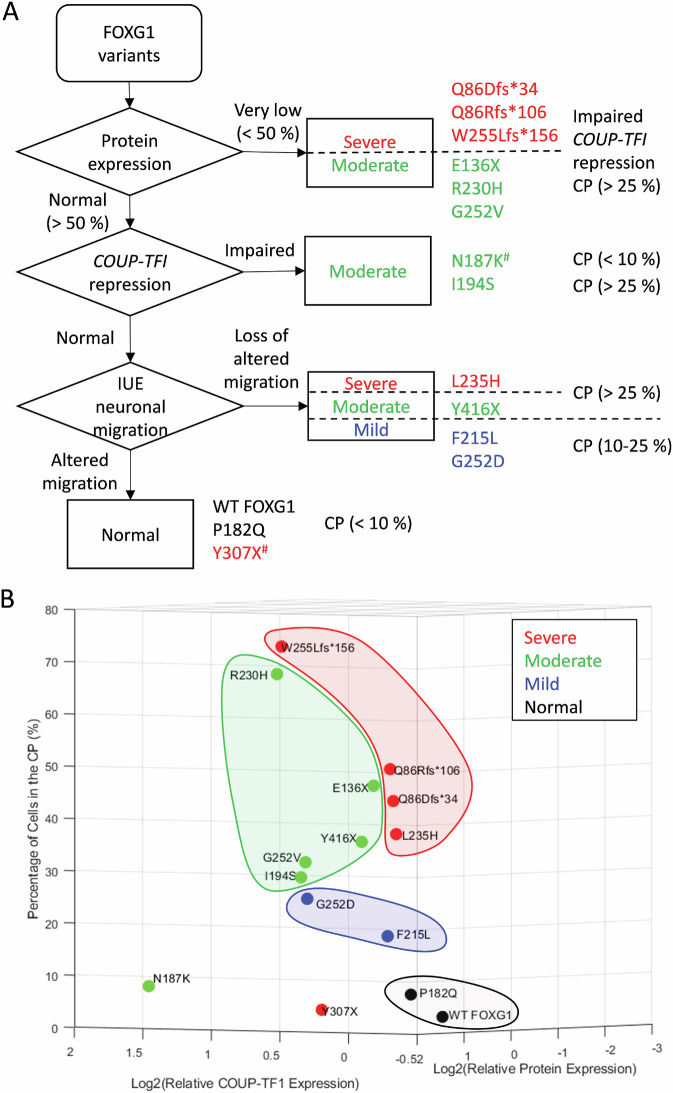
A flowchart for predicting the severity of brain anomalies caused by *FOXG1* variants through in vitro and in vivo assays. **A** The expression levels of FOXG1 variants are first examined, and variants with very low expression (<50%) tend to cause severe to moderate brain anomalies. This categorization can be further refined through the *COUP-TFI* repression assay. While all very low expression variants exhibit impaired repression, among variants with normal to high expression levels, those showing impaired *COUP-TFI* repression tend to lead to moderate brain anomalies. For *FOXG1* variants demonstrating relatively normal *COUP-TFI* repression ability, their effects on neuronal migration and differentiation are further assessed through IUE. In cases of WT FOXG1 and variants that are associated with normal MRI, fewer than 10% of cells typically reach the CP. Variants leading to severe and moderate brain anomalies usually result in a higher percentage of cells (>25%) reaching the CP, while mild variants lead to 10–25% of cells reaching the CP. It’s worth noting that among these variants, only Y307X, which is associated with severe brain anomalies, does not align with our prediction model. #: variants that did not entirely fit the model. **B** PCA of FOXG1 variant severity. Data from protein expression, *COUP-TFI* repression, and neuronal migration assays were plotted in a three-dimensional scatter plot, with severity groups color-coded. Clustering analysis effectively differentiates normal, mild, moderate, and severe brain anomalies, with the exception of N187K and Y307X, which remain outliers.

Based on these functional assays, we developed a flowchart to predict the severity of brain anomalies resulting from *FOXG1* variants (Fig. 5A). *FOXG1* variants initially classified as very low expressers (<50% of WT expression) typically lead to severe (Q86Dsf*34, Q86Rfs*106, and W255Lfs*156) or moderate (E136X, R230H, and G252V) brain anomalies. This assay identified 54.5% (6/11) of moderate-to-severe cases and no (0/2) mild cases. Further refinement is achieved through a *COUP-TFI* repression assay. For variants with normal or high expression levels, those exhibiting impaired *COUP-TFI* repression ability (N187K and I194S) resulted in moderate brain anomalies, increasing the sensitivity of detecting moderate-to-severe cases from 54.5% (6/11) to 72.7 (8/11). *FOXG1* variants demonstrating relatively normal *COUP-TFI* repression ability should undergo a further assessment of their impact on neuronal migration through IUE. In cases of WT and variants leading to normal MRI (P182Q), fewer than 10% of cells typically reach the CP. Variants leading to severe (L235H) and moderate (Y416X) brain anomalies usually allow a higher percentage of cells (>25%) to reach the CP, while mild variants (e.g., F215L and G252D) result in 10–25% of cells reaching the CP. Among these variants, only Y307X, associated with severe brain anomalies, does not align with our prediction model. Combining all three assays resulted in a sensitivity of 90.9% (10/11) for moderate-to-severe cases, 100% (2/2) for mild cases, and an overall sensitivity of 92.3% (12/13).

Interestingly, there are only moderate correlations between these 3 assays (Supplementary Fig. 5), suggesting each functional assay provides valuable insights into the properties and functions of different variants. To further validate our workflow, we applied principal component analysis (PCA) to visualize the clustering of variants based on FOXG1 protein expression, *COUP-TFI* repression in the luciferase assay, and the percentage of cells reaching the CP in the migration assay. This analysis revealed a clear separation of normal, mild, moderate, and severe MRI phenotypes, with the exception of N187K and Y307X (Fig. 5B), reinforcing the robustness of our predictive framework.

## Discussion

In this study, we investigated the impact of FOXG1 variants on brain development to predict the severity of FOXG1 syndrome. We identified a correlation between the molecular and cellular effects of FOXG1 mutations and brain malformations rather than the clinical severity score, as clinical severity may be influenced by age, broader neurological functions, and other factors beyond cortical development. By assessing clinical symptoms, brain MRI findings (Table 1), protein expression levels (Fig. 1), *COUP-TFI* repression (Fig. 2), neuronal migration (Fig. 3), and differentiation (Fig. 4) associated with different *Foxg1* variants, we developed a workflow to predict the severity of brain anomalies (Fig. 5). This approach accurately identified 90.9% (10/11) of cases with moderate-to-severe brain anomalies and correctly distinguished all mild and normal cases (3/3). This diagnostic workflow could prove valuable for predicting the pathogenicity of newly identified *FOXG1* variants through NGS during prenatal or neonatal genetic screening.

The *FOXG1* variants observed in FOXG1 syndrome display diverse prognoses (Table 1) [18–20]. Protein expression analysis provided insights into the functional impacts of these variants (Fig. 1). FOXG1 is initially expressed in cortical progenitor cells (E8.5–E10.5) and later in neurons (E13) [4]. Once expressed, FOXG1 promotes cortical progenitor proliferation and guides their differentiation into projection neurons while orchestrating the sequential generation of deep-layer (DL) and upper-layer (UL) neurons [13]. FOXG1 functions in a spatiotemporal manner, as demonstrated by its suppression by EGR2 in early postmitotic stages, which leads to COUP-TFI upregulation and facilitates L4 neuron specification [7]. In our scRNA-seq analysis at E13.5 and E15.5, *Foxg1* expression was predominantly observed in neurons, with lower levels in progenitors and precursors (Fig. 2B). This pattern, along with previous studies, suggests potential differences in the temporal dynamics of FOXG1 mRNA and protein expression across developmental timepoints. Interestingly, pathogenic variants with very low expression were generally associated with moderate to severe brain anomalies, most of which also impaired *COUP-TFI* repression (Fig. 2), indicating disruptions in FOXG1’s DNA binding function and its regulation of target genes. Notably, some variants with normal protein levels still impaired *COUP-TFI* repression, emphasizing that protein expression alone does not determine functional outcomes.

FOXG1 overexpression dramatically altered neuronal distribution (Fig. 3) and the expression of layer markers (Fig. 4), consistent with its role in regulating neuronal migration and cell fate [3]. Disease-associated *Foxg1* variants with very low protein expression all resulted in cell distributions similar to those in brains electroporated with the empty vector. Among the variants with normal expression but LOF in *COUP-TFI* repression, I194S exhibited LOF in altering neuronal migration, whereas N187K delayed neuronal migration similar to WT FOXG1. This inconsistency may be caused by the compensatory effect of the N187K variant acting on other downstream targets critical for neuronal migration in mice. Nevertheless, N187K caused moderate brain anomalies in humans, correctly correlated with LOF in *COUP-TFI* repression, emphasizing the importance of the COUP-TFI repression assay. Among the variants that showed neither very low expression nor LOF in *COUP-TFI* repression, their ability to alter neuronal migration correlated well with the severity of brain anomalies (Figs. 3, 5). Intriguingly, the nonsense variant Y307X, associated with severe brain anomalies, showed no defects in any of these functional assays, suggesting that additional assays are needed to more accurately predict clinical severity based on functional loss.

With the increase use of NGS, whole exome sequencing (WES) is frequently employed to identify genetic causes of neurological disorders, especially when a genetic origin is suspected. Some parents opt for prenatal or neonatal NGS screening to assess genetic disorder risks, but distinguishing between benign and pathogenic variants remains a challenge. While ongoing efforts aim to refine methodologies using databases and software tools, our study proposes a cost-effective approach to predict possible pathogenic variants of *FOXG1*. The three assays can be performed simultaneously within a two-week timeframe, offering valuable tools for early diagnosis and treatment stratification. This workflow could also estimate the probability of brain anomalies for individuals carrying variants that do not show abnormality in all three assays (currently with a false-negative rate of 7.69%). However, the IUE assay, in particular, requires specialized expertise and equipment that may not be readily available in diagnostic laboratories. Future studies should focus on developing alternative, more clinically accessible approaches that retain the core functional insights provided by these assays while facilitating broader implementation in clinical practice.

A promising application of these assays is to stratify individuals for early treatment. A recent study demonstrated the effectiveness of a CRISPR/Cas9 system coupled with adeno-associated virus (AAV) in repairing *FOXG1* variants in human cell lines, including hiPSCs [42]. This approach normalized PAX6 expression levels in developing neurons, laying the foundation for novel personalized therapies for FOXG1 syndrome. Despite challenges in applying treatments due to FOXG1’s critical role during early brain development, early prediction of potential variant effects may assist in risk-benefit assessments for early gene therapy in FOXG1 syndrome.

### Study limitations

This study has several limitations that should be acknowledged. First, the sample size is small, comprising only 14 individuals with FOXG1 syndrome, which is a consequence of the disorder’s rarity. The wide age range of the participants (14–263 months) introduces variability that may influence clinical severity scores. Additionally, due to the small sample size, multiple testing corrections, such as Bonferroni or FDR, were not applied, as they would further reduce statistical power. Consequently, the correlation coefficients (R² values) between functional assays and clinical severity were relatively low. Another limitation is that only a single MRI series was available for most individuals collected from the literature, which may affect the consistency of imaging-based severity assessments. Furthermore, although we identified a strong correlation between FOXG1 variants and brain malformations, the relationship with broader clinical phenotypes-including somatic growth, motor and speech development, behavior, and neurological features-remained less defined, suggesting that FOXG1 may exert additional functions at different developmental stages. Lastly, due to cost constraints, all experiments were performed in triplicate, and while ANOVA was used for statistical analysis, larger replication studies are needed to strengthen the robustness of these findings. Despite these limitations, our proposed workflow provides a valuable framework for predicting the severity of brain anomalies in FOXG1 syndrome and may contribute to future diagnostic and therapeutic strategies.

## Supplementary information

Supplemental table

Supplemental figures

## Data Availability

All data contained in this study are available from the corresponding author upon reasonable request.
