# Supplementary material for: Functional defects in FOXG1 variants predict the severity of brain anomalies in FOXG1 syndrome
Source: Mol Psychiatry. 2025 Jun 16;30(10):4824–35. doi: 10.1038/s41380-025-03077-y (PMC12436187; doi:10.1038/s41380-025-03077-y)
Supplement: Supplementary file 1 — Supplemental table [file 41380_2025_3077_MOESM1_ESM.docx]

| MRI feature | Score |  | |  |  |
| --- | --- | --- | --- | --- | --- |
|  | 0 | 1 | | 2 | 3 |
| Simplified gyral pattern | Absent | | Present |  |  |
| Basal ganglia | Normal | | Hypoplasia |  |  |
| Inner CSF spaces | Normal | | Enlarged |  |  |
| Corpus callosum | Normal | | Thinning | Partial agenesis | Complete agenesis |
| Frontal lobes | Normal | | Hypoplasia |  |  |

Supplementary Table 1. FOXG1 brain MRI severity score (adapted and modified based on the work of M. Pringsheim et al.^1^.)

A score of 0 indicated completely normal MRI results, and a score of 6 indicated the most severe neuroimaging anomaly, scores between 0 and 2 indicated normal to mild severity, scores of 3 and 4 signified moderate severity, and scores in the range of 5 to 6 represented severe severity.

1. Pringsheim M, Mitter D, Schröder S, et al. Structural brain anomalies in patients with FOXG1 syndrome and in Foxg1+/- mice. *Annals of clinical and translational neurology*. Apr 2019;6(4):655-668. doi:10.1002/acn3.735
