# Supplementary material for: Functional defects in FOXG1 variants predict the severity of brain anomalies in FOXG1 syndrome
Source: Mol Psychiatry. 2025 Jun 16;30(10):4824–35. doi: 10.1038/s41380-025-03077-y (PMC12436187; doi:10.1038/s41380-025-03077-y)
Supplement: Supplementary file 2 — Supplemental figures [file 41380_2025_3077_MOESM2_ESM.pdf]

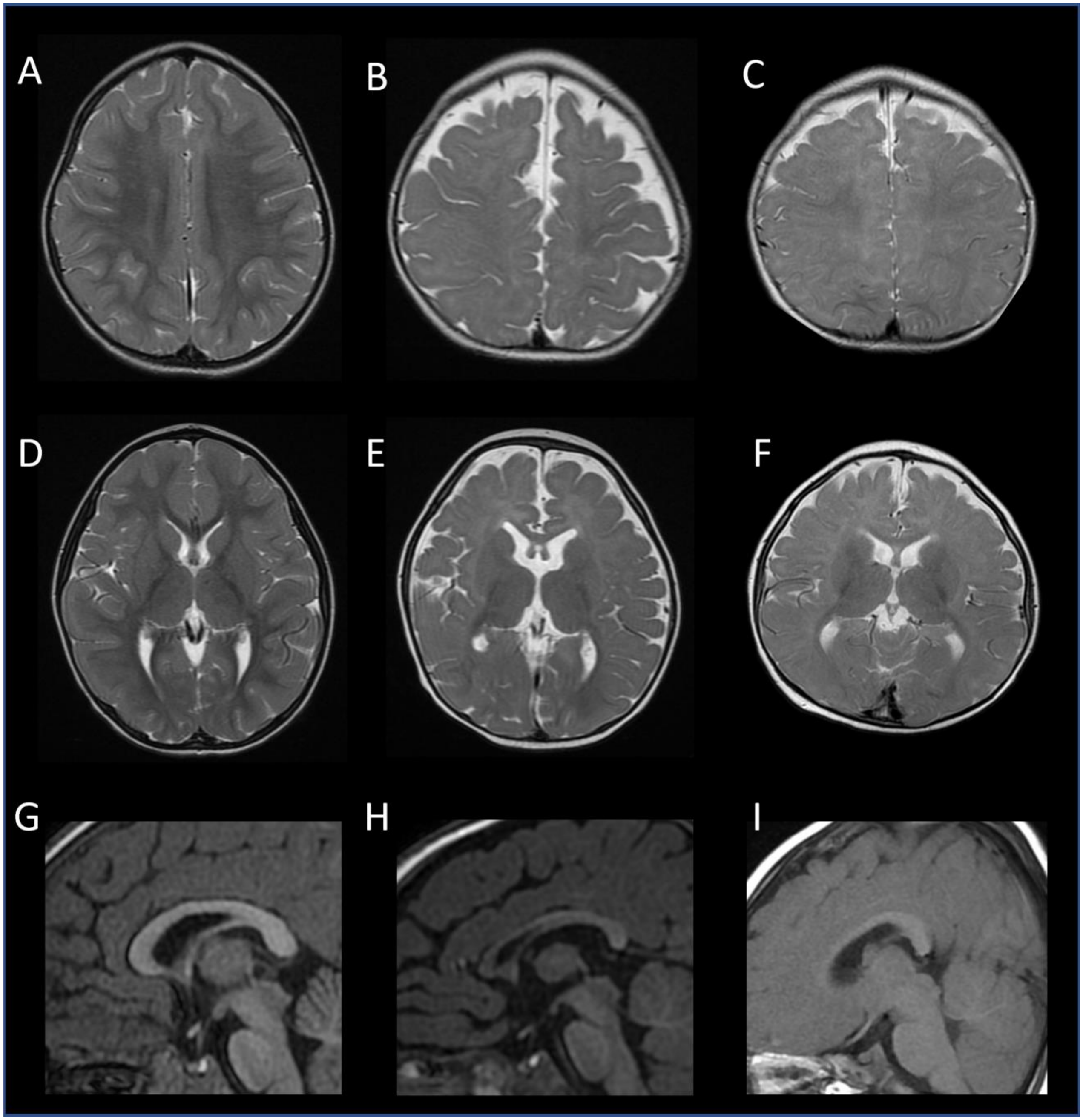

Supplementary Figure 1. The range of structural brain anomalies observed in FOXG1 syndrome, as depicted through brain MRI scans. A–C show gyral pattern of the fronto-parietal area in three patients with FOXG1 syndrome in T2-weighted axial MRI: A (patient 7), B (patient 1), C (patient 9) exhibit mild to moderate simplified gyral pattern, with dilated subarachnoid CSF spaces (B, C). D-F show basal ganglia in T2-weighted axial MRI: D (patient 7) normal basal ganglia, E &F (patient 1, patient 9) small basal ganglia relative to thalamus, with dilated ventricles. G-I show corpus callosum (CC) in T1-weighted midsagittal MRI : G (patient 7) normal CC, relative thinning (H, patient1) and partial agenesis of anterior parts to (I, patient 9)

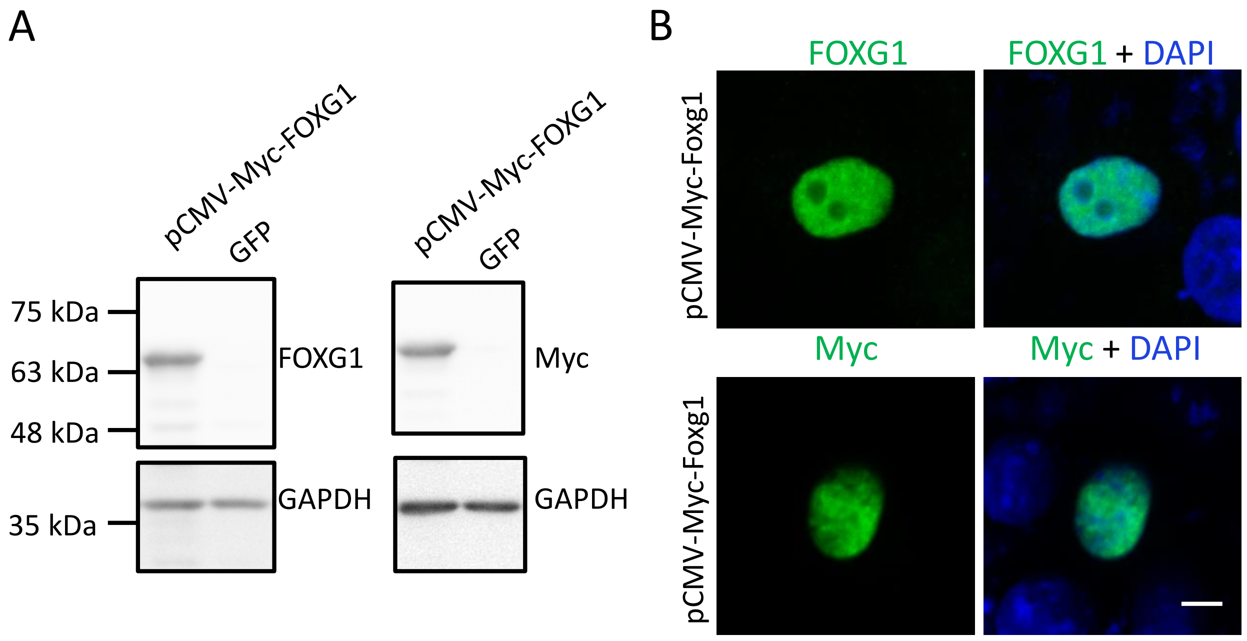

Supplementary Figure 2. The expression profile of the pCMV-Myc-Foxg1 construct. (A) The protein expression of FOXG1 using FOXG1 and Myc antibodies. The construct was transfected to HEK293T cells for 24hr. Protein expression was verified by western blotting. (B) Subcellular localization of FOXG1 (green) in HEK293T cells transfected with pCMV-Myc-FOXG1. Cells were immunostained with FOXG1 and Myc antibodies and counter stained with DAPI (blue). Bar = 10 µm.

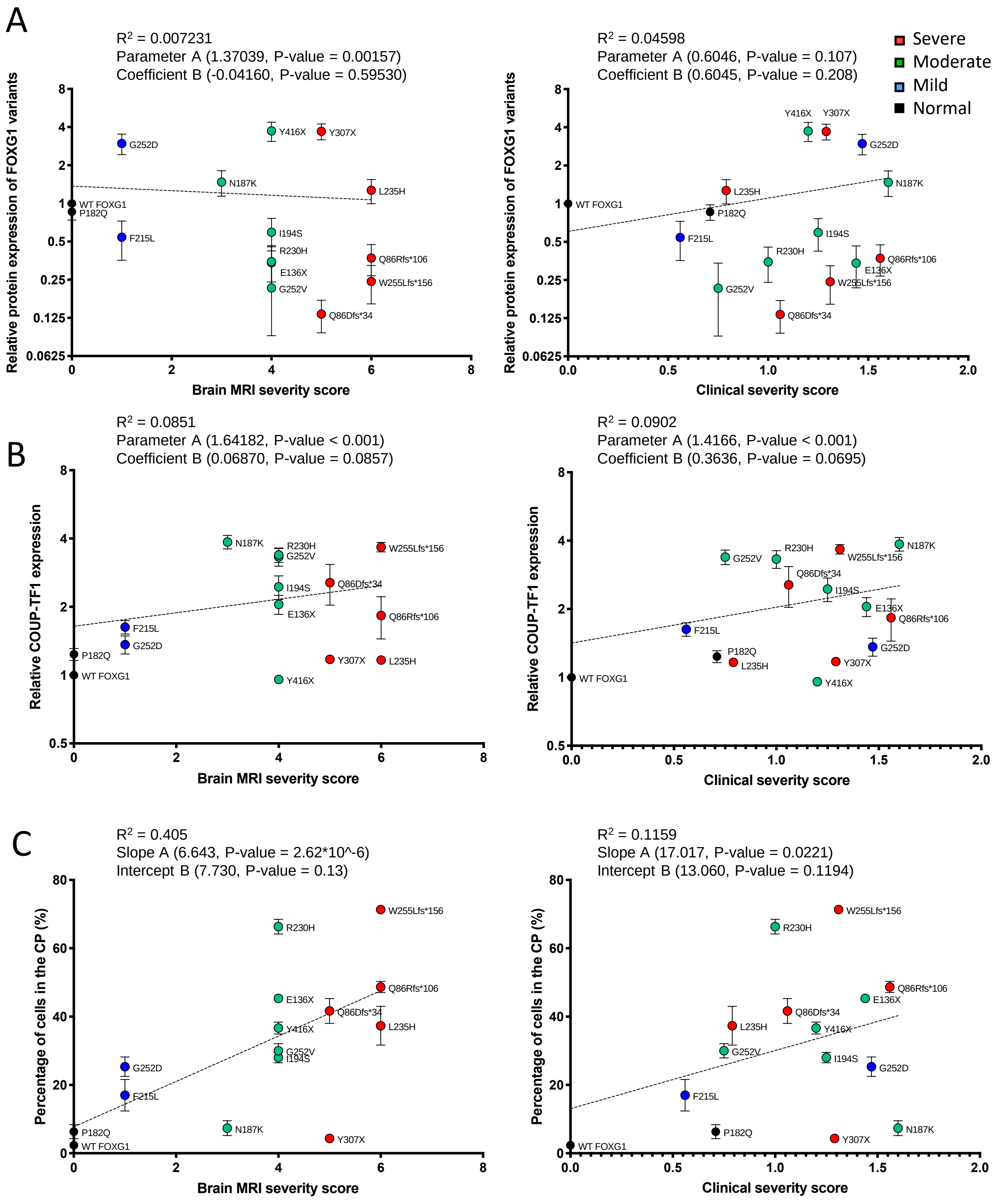

Supplementary Figure 3. Correlations between brain MRI severity, clinical severity scores, and FOXG1 functional assays. (A) The protein expression levels of FOXG1 variants exhibit weak correlations with both brain MRI severity ($R^2 = 0.0072$) and clinical severity scores ($R^2 = 0.046$). Neither correlation is statistically significant ($p = 0.595$ and $p = 0.208$, respectively), indicating a minimal or negligible effect. (B) The relative expression of *COUP-TFI* under FOXG1 regulation shows weak correlations with both severity scores ($R^2 = 0.085$ and $R^2 = 0.090$, respectively). While these correlations do not reach statistical significance ($p = 0.0857$ and $p = 0.0695$, respectively), they trend toward significance, suggesting a potential contribution. (C) The percentage of cells reaching the CP exhibits a moderate positive correlation with brain MRI severity ($R^2 = 0.405$), with a statistically significant slope (6.653, $p = 2.62 \times 10^{-6}$). In contrast, the correlation with clinical severity scores is weaker ($R^2 = 0.116$) but remains statistically significant (Slope = 17.017, $p = 0.0221$). Error bars represent SEM.

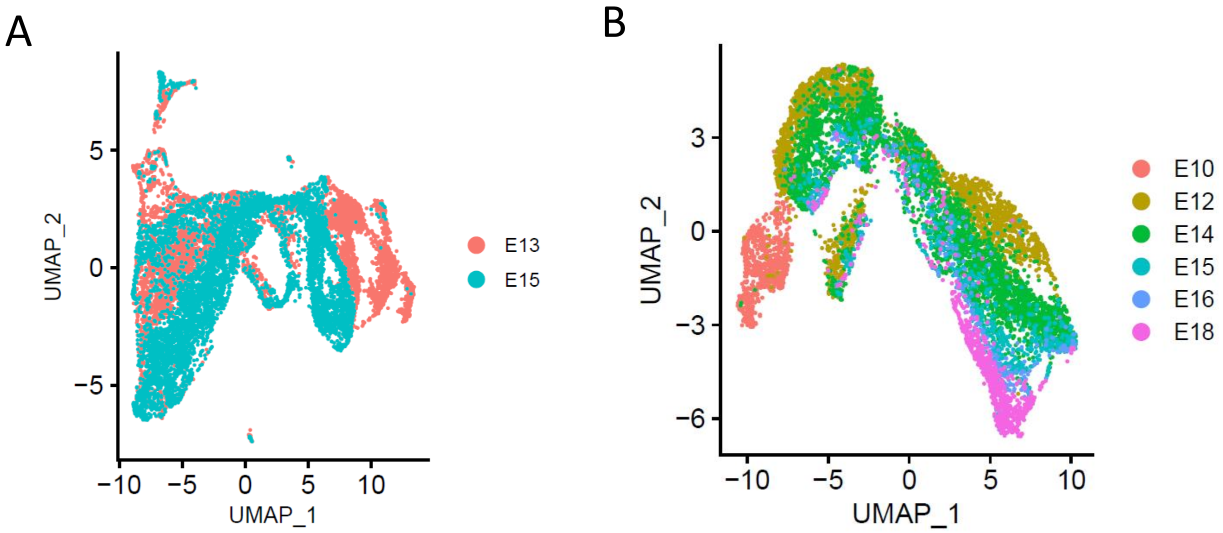

Supplementary Figure 4. UMAP plots showing the cellular origin of scRNA-seq data from mouse embryos at various developmental stages, corresponding to Figure 2A-C. (A) Cells from mouse cortices at E13.5 and 15.5 (B) Cells from mouse cortices at E10, 12, 14, 15, 16 and 18.

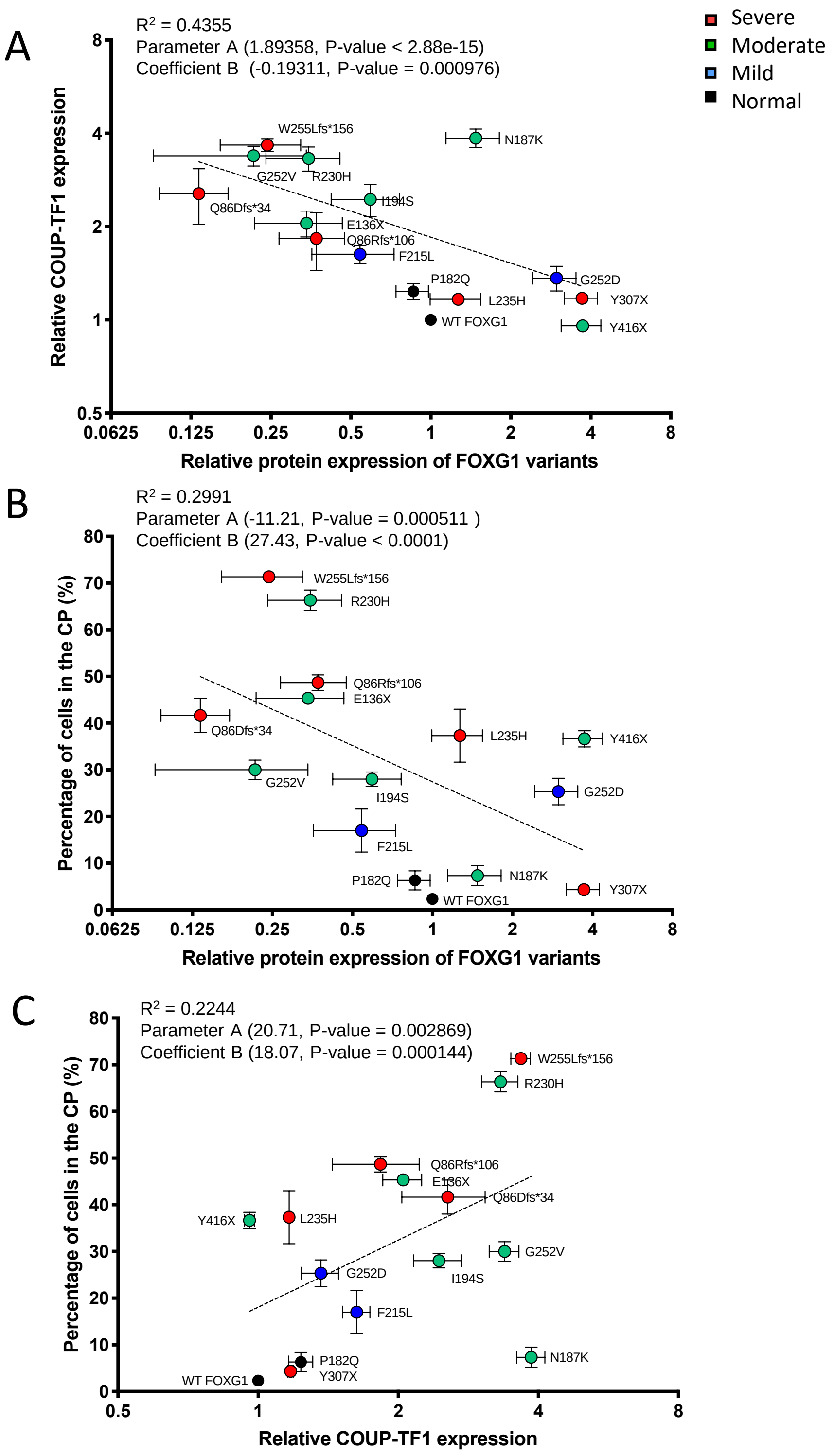

Supplementary Figure 5. The correlation among FOXG1 expression, *COUP-TFI* repression, and neuronal migration assays. (A) *COUP-TFI* expression under FOXG1 regulation exhibits a moderate positive correlation with FOXG1 protein expression levels (R^2 = 0.4355), with a statistically significant association (p = 0.000976). (B) The percentage of cells reaching the CP negatively correlates with FOXG1 protein expression levels (R^2 = 0.2991), indicating that higher FOXG1 expression is associated with impaired neuronal migration. This relationship is statistically significant (p = 0.000511) .(C) The percentage of cells reaching the CP positively correlates with *COUP-TFI* expression under FOXG1 control (R^2 = 0.2244), suggesting that reduced *COUP-TFI* repression by FOXG1 variants may contribute to neuronal migration deficits. This correlation is statistically significant (p = 0.002869). Error bars represent SEM.
